# Saponification Value of Fats and Oils as Determined from ^1^H-NMR Data: The Case of Dairy Fats

**DOI:** 10.3390/foods11101466

**Published:** 2022-05-18

**Authors:** Mihaela Ivanova, Anamaria Hanganu, Raluca Dumitriu, Mihaela Tociu, Galin Ivanov, Cristina Stavarache, Liliana Popescu, Aliona Ghendov-Mosanu, Rodica Sturza, Calin Deleanu, Nicoleta-Aurelia Chira

**Affiliations:** 1 Department of Milk and Dairy Products, Technological Faculty, University of Food Technologies, 26 “Maritsa” Blvd., 4002 Plovdiv, Bulgaria; mivanova@uft-plovdiv.bg (M.I.); ivanovgalin.uft@gmail.com (G.I.); 2 Department of Organic Chemistry, Biochemistry and Catalysis, Research Centre of Applied Organic Chemistry, Faculty of Chemistry, University of Bucharest, 90-92 Panduri Street, 050663 Bucharest, Romania; anamaria_hanganu@yahoo.com; 3 “C.D. Nenitescu” Centre of Organic Chemistry of the Romanian Academy, 202B Spl. Independentei, 060023 Bucharest, Romania; crisstavarache@gmail.com (C.S.); calin.deleanu@yahoo.com (C.D.); 4 “C.D. Nenitescu” Organic Chemistry Department, Faculty of Chemical Engineering and Biotechnologies, University POLITEHNICA of Bucharest, 1-7 Polizu Street, 011061 Bucharest, Romania; ralu_dumitriu@yahoo.com (R.D.); mihaela.tociu@upb.ro (M.T.); 5 Advanced Polymer Materials Group, University POLITEHNICA of Bucharest, 1-7 Gh. Polizu Street, 011061 Bucharest, Romania; 6 Department of Oenology and Chemistry, Food Technology, Faculty of Food Technology, Technical University of Moldova, 9/9 Studentilor Street, MD-2045 Chisinau, Moldova; liliana.popescu@tpa.utm.md (L.P.); aliona.mosanu@tpa.utm.md (A.G.-M.); rodica.sturza@chim.utm.md (R.S.); 7 “Petru Poni” Institute of Macromolecular Chemistry of the Romanian Academy, Aleea Grigore Ghica Voda 41A, 700487 Iasi, Romania

**Keywords:** saponification value, ^1^H-NMR spectroscopy, tributyrin, dairy fat, vegetable oils

## Abstract

The saponification value of fats and oils is one of the most common quality indices, reflecting the mean molecular weight of the constituting triacylglycerols. Proton nuclear magnetic resonance (^1^H-NMR) spectra of fats and oils display specific resonances for the protons from the structural patterns of the triacylglycerols (i.e., the glycerol backbone), methylene (-CH_2_-) groups, double bonds (-CH=CH-) and the terminal methyl (-CH_3_) group from the three fatty acyl chains. Consequently, chemometric equations based on the integral values of the ^1^H-NMR resonances allow for the calculation of the mean molecular weight of triacylglycerol species, leading to the determination of the number of moles of triacylglycerol species *per* 1 g of fat and eventually to the calculation of the saponification value (SV), expressed as mg KOH/g of fat. The algorithm was verified on a series of binary mixtures of tributyrin (TB) and vegetable oils (i.e., soybean and rapeseed oils) in various ratios, ensuring a wide range of SV. Compared to the conventional technique for SV determination (ISO 3657:2013) based on titration, the obtained ^1^H-NMR-based saponification values differed by a mean percent deviation of 3%, suggesting the new method is a convenient and rapid alternate approach. Moreover, compared to other reported methods of determining the SV from spectroscopic data, this method is not based on regression equations and, consequently, does not require calibration from a database, as the SV is computed directly and independently from the ^1^H-NMR spectrum of a given oil/fat sample.

## 1. Introduction

One of the most common oil quality indices is the saponification value (SV); it is defined as the amount of alkali (expressed as mg KOH/g sample) required to saponify a defined amount of sample. It is conventionally determined through saponification of a known amount of oil/fat with excess KOH solution, followed by back titration of the excess base with acid solution in the presence of phenolphthalein as an indicator. The amount of base needed for saponification of the fatty acyl chains is then indirectly determined from the excess base that remains unreacted. Since the amount (moles) of base reacted is stoichiometrically equal to the amount (moles) of fatty acyl chains contained in 1 g of oil/fat, SV is then dependent on the length of the fatty acyl chains from triacylglycerols. Therefore, a small saponification value indicates long chain fatty acids on the glycerol backbone in a sample; on the contrary, a high SV indicates triacylglycerols with shorter fatty acyl chains. Consequently, SV becomes an easy approach to assess fatty acids’ chain length of specific fats/oils. 

For example, most of the common oils/fats of vegetable or animal origin (sunflower, soybean, rapeseed, pork lard, beef tallow, chicken fat, etc.) contain almost only long chain fatty acids (C18 and C16), having similar SV values (ranging from 168–196 mg KOH/g oil) [1]. Some vegetable oils, such as the coconut and palm kernel oils, contain large amounts of lauric (C12:0) and myristic (C14:0) acids; therefore, their saponification values are significantly higher (235–260 mg KOH/g oil) [2,3,4,5]. Milk fat differs substantially from other fats and oils in terms of the fatty acid profile (FAP), including relevant amounts of short chain (C4–C6) and medium chain (C8–C12) fatty acids, which is subsequently reflected in its high SV (213–227 mg KOH/g fat) [6,7]. Consequently, SV may be helpful in the detection of the adulteration of dairy products with cheaper fats and oils, because the addition of an oil/fat rich in C18 to a dairy product will result in a decrease in the SV.

Although easy and accurate, the reference method of SV determination requires specific glassware and harmful chemicals and is time consuming (according to the protocol, the saponification step takes one hour to complete, because it is critical that the saponification be complete prior to the final titration). In addition, several factors can cause errors in the titration step including misjudging the color of the indicator near the end point, misreading volumes or faulty technique. Therefore, a new, rapid and reliable method would be preferred.

In this respect, spectroscopic methods coupled with multivariate data analysis have attracted attention, being considerably faster and more practical from a procedural viewpoint. For example, SV has been determined through Fourier transform infrared spectroscopy (FTIR) coupled with multivariate analysis [8] with good accuracy, compared to the standard method; however, the main drawback of the methods based on spectroscopic data is that they require the existence of a large spectral base for the model calibration.

^1^H-NMR spectroscopy is a fast (the recording of a ^1^H-NMR spectrum takes approximately 2 min) and non-destructive technique that has widely been applied in the analysis of edible oils. ^1^H-NMR spectra of fats and oils display signals assigned to both the unsaturated moiety and to various methylene groups of the fatty acyl chains. These signals may be used to calculate the average fatty acyl chain length of fat samples. The ^1^H-NMR technique allows for full process automation, from the recording (due to the autosamplers) to data processing. Small amounts of samples are necessary, which—if needed—can further be recovered simply through solvent evaporation, after the spectra are recorded. Very importantly, the ^1^H-NMR technique is also reliable, and several papers report the fatty acid profile of fats and oils computed from ^1^H-NMR data in good agreement with chromatographic data [9,10,11,12,13]. Skiera et al. briefly reported a rapid method for the determination of the SV from NMR data based on the integral of the CH_2_ protons adjacent to the ester groups (δ_H_ 2.2–2.4 ppm) and on the integral of the 1,2,4,5-tetrachloro-3-nitrobenzene (TCNB) signal at δ_H_ 7.7 ppm, used as an internal standard for quantitative NMR experiments. Five samples (with a single measurement *per* sample) were tested with the new method; the NMR results were in agreement with the values obtained through the ISO method, consequently pointing at the suitability of the NMR spectroscopy for the determination of the quality indices of fats and oils [14].

Based on our previous expertise on NMR chemometrics to edible oils [13], the present work reports a general algorithm for the calculation of the SV of fats and oils from the ^1^H-NMR data. The working model consists of a series of binary mixtures of tributyrin (TB) and vegetable oils in various ratios to obtain a wide range of SV. In addition, to ensure an even more variate composition also regarding the unsaturation, soybean and rapeseed oils—SO and RO, respectively—were used to prepare the model samples. The average length of the fatty acyl chains can be computed through chemometric equations from ^1^H-NMR data, leading to the calculation of the average molecular weight of each sample and eventually to the SV. The new method was evaluated in comparison with the conventional method based on titration and was further applied to a series of edible fats and oils including butter and cheese extracted fats. Compared to other reported methods of determining the SV from spectroscopic data, the proposed method is not based on regression equations and, consequently, does not require calibration from a database. SV may be computed directly and independently from the ^1^H-NMR spectrum of a given oil/fat sample.

## 2. Materials and Methods

### 2.1. Reagents

CH_2_Cl_2_ (HPLC purity) and anhydrous MgSO_4_ were from Sigma–Aldrich, as well as tributyrin (97%). The CDCl_3_ (isotopic purity 99.8%D) was also from Sigma–Aldrich.

### 2.2. Binary Oil–Tributyrin Mixtures

A series of binary mixtures of tributyrin (TB) and vegetable oils (RO and SO) in various ratios was prepared to obtain a wide range of SVs. Owing to their different fatty acid profiles, SO and RO were chosen as components for binary mixtures to obtain an even more variate composition also with respect to the unsaturation, thus leading to more reliable results. The specific composition of the RO-TB and SO-TB series is given in the Supplementary Table S1.

### 2.3. Butter and Cheese Samples

Butter (*n* = 4) and cheese (*n* = 9) samples of bovine origin were obtained from Romanian, Bulgarian and Moldavian dairy companies. Butter fat (BF) was extracted from butter samples with CH_2_Cl_2_, dried on anhydrous MgSO_4_, followed by evaporation of the solvent. Cheese fat was extracted according to ISO 1735|IDF 5:2004 protocol [15].

### 2.4. Oil and Fat Samples

Soybean, rapeseed and sunflower seeds were obtained from the National Agricultural Research and Development Institute of Fundulea (NARDI Fundulea), Romania. The oil was extracted from seeds according to the standard Soxhlet protocol [16]. Beef and sheep tallow were extracted with CH_2_Cl_2_ from subcutaneous adipose tissue, dried on anhydrous MgSO_4_, followed by evaporation of the solvent. Coconut oil was purchased from Trio Verde S.R.L., Romania (distributor), and the palm stearin and palm kernel oil were from Scintilla Silk, Romania (distributor).

### 2.5. Saponification Value

The saponification value was determined according to the ISO 3657:2013 standard procedure [17].

### 2.6. ^1^H-NMR Spectra

^1^H-NMR experiments were recorded in a field of 6.9 T using a Bruker Fourier spectrometer (Bruker Biospin, Ettlingen) operating at an ^1^H Larmor frequency of 300.18 MHz. The ^1^H-NMR experiments were using the standard zg30 pulse sequence and had the following parameters: 30° pulse, 5.37 s acquisition time, 6.1 kHz spectral window, 16 scans, 65K data points, 1 s delay time; all spectra were recorded at 25 °C. Fat samples (200 mg) were dissolved in 0.6 mL CDCl_3_ and transferred to 0.5 mm NMR tubes of the type Norell NOR508UP7-5EA (Sigma–Aldrich, Saint Louis, MO, USA). MestReNova 6.0.2-5475 software (Mestrelab Research, Santiago de Compostela, Spain) was used to process the spectra.

To eliminate operator errors, fixed integration limits were used to obtain the integration values (Supplementary Materials Table S1). In addition, for each sample the F resonance (given by the two protons adjacent to the ester group) was considered as a reference and, therefore, calibrated to 2.000; consequently, the rest of the integrals were automatically reported to the reference. According to the general rule for signals integration (i.e., from baseline to baseline), partially overlapping signals were integrated altogether (i.e., A + B and I + J, respectively). The NMR tubes were in-house quality checked as we previously reported [18].

### 2.7. Statistics

The experiments were run in triplicate (NMR) and in duplicate (ISO 3657:2013). The results are expressed as the mean values ± standard deviation (sd). Tuckey’s test was applied for the significantly different means (*p* < 0.05).

## 3. Results and Discussions

### 3.1. ^1^H-NMR Spectral Characterization of Fats and Oils

**Figure 1:**
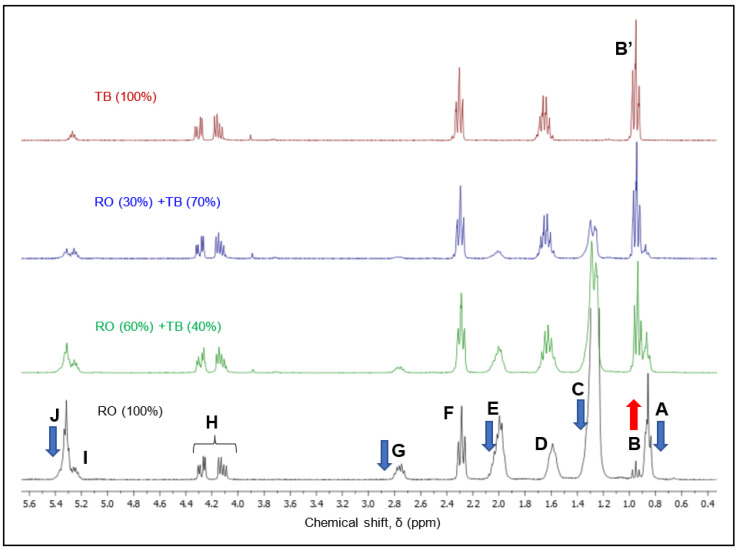
Comparative ^1^H-NMR spectral characterization of tributyrin (TB ―), rapeseed oil (RO ―) and rapeseed oil–tributyrin binary mixtures: RO (30%) + TB (70%) ― and RO (60%) + TB (40%) ―. Letters A–J were assigned to resonances according to letters in Table 1.

A typical ^1^H-NMR spectrum of an oil is illustrated for a rapeseed oil (RO) in Figure 1. The corresponding peak assignment is explained in Table 1. Figure 1 also shows a comparison of the ^1^H-NMR spectra of tributyrin (TB) and two rapeseed oil–tributyrin binary mixture: RO (30%) + TB (70%) and RO (60%) + TB (40%).

As reflected from Figure 1, certain signals (i.e., A, C, E and J) cannot be found in the spectrum of tributyrin, because butyric acid is a short chain saturated fatty acid, lacking allylic, bis-allylic and unsaturated protons. The butyric moiety displays the triplet B’ characteristic of the terminal methyl group in the structure of fatty acids, the signal D of the protons in position β relative to the ester group, the triplet F generated by the methylene groups adjacent to the ester group and the signals in the specific area of the glycerol backbone (H and I). We have previously shown the assignment of NMR signals in methyl esters of fatty acids as standards for vegetable oil characterization [20]. We have also shown [19] that the resonance characteristic to the terminal methyl group of the fatty acyl chains appears shifted downfield (0.96 ppm) only in the case of linolenic and butyric acyl moieties (B and B’, respectively), compared to the rest of the fatty acyl chains (triplet A, 0.85 ppm). It is therefore evident that as the amount of TB added to the vegetable oil increases, all the resonances related to unsaturated specific groups (J) and those in the vicinity of allylic and bis-allylic groups, (E and G) will decrease. The amplitude of signal C also decreases with the addition of TB, as this resonance is dependent on the length of the fatty acyl chains, being absent for TB.

The only signal that increases in intensity is the triplet B from 0.96 ppm, characteristic for the terminal methyl group in butyric acid or linolenic acid. In rapeseed oil, the 0.96 ppm resonance is due to the linolenic acyl moiety (signal B); as the percentage of added TB increases, this resonance also increases in intensity due to the overlapping signal B’. As expected, the unspecific signals present in all fats and oils, regardless of their specific fatty acid profile (such as H and I from the glycerol moiety, as well as D and F adjacent to the ester group), did not show modifications.

### 3.2. Algorithm for the SV Calculation from ^1^H-NMR Data

**Figure 2:**
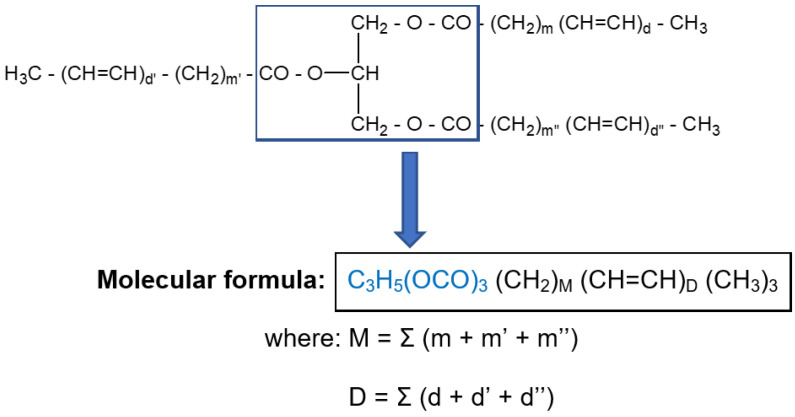
General representation of a triacylglycerol structure.

The general pattern of triacylglycerols (TAGs), as depicted in Figure 2, consists of a glycerol ester backbone and three fatty acyl chains, each with a terminal methyl group and various amounts of methylene and CH=CH double bonds.

As reflected from Figure 2, triacylglycerols consist of a glycerol triple ester backbone, common to all TAGs, the differences occurring in the hydrocarbon residues from fatty acyl chains. Apart from the terminal methyl groups (-CH_3_), the hydrocarbon chains consist only of methylene groups (-CH_2_-) and double bonds (-CH=CH-), the number of which differs depending on the length of the chain and on the degree of unsaturation, being characteristic for each individual fatty acid. For example, oleic acid contains fourteen methylene groups (-CH_2_-) and a single double bond (-CH=CH-), and linoleic acid contains twelve methylene groups (-CH_2_-) and two double bonds (-CH=CH-). Therefore, the average molecular formula of a triglyceride can be rendered as:

C_3_H_5_(OCO)_3_(CH_2_)_M_(CH=CH)_D_(CH_3_)_3_

The integral of a resonance being the area under the resonance curve, in the next chemometric equations the following suggestive notations were adopted for the integral values of the corresponding resonances: A_(A+B)_, A_C_, A_D_, A_E_, A_F_, A_G_, A_H_, and A_(I+J)_, respectively.

The average number of methylene groups (M) and the average number of double bonds (D) in the alkyl chain can then be calculated as:

(1) $$M=\frac{3}{2}\cdot \frac{A_{C}+A_{D}+A_{E}+A_{F}+A_{G}}{A_{\left(A+B\right)}}$$

(2) $$D=\frac{3}{2}\cdot \frac{A_{\left(I+J\right)}-A_{H}/4}{A_{\left(A+B\right)}}$$

(i) The normalization factor 3/2 appeared as a consequence of the different number of protons that generated the resonances involved in Equations (1) and (2), i.e., two protons in the case of the resonances at the numerator and three in the case of the resonances at the denominator;
(ii) Since resonances I and J appear partially overlapped, they cannot be integrated separately. However, A_I_ (corresponding to the single proton in the sn-2 position from the glycerol moiety) can be indirectly computed as $A_{H}/4$, given the proton ratio of 1:4 in the case of signals I and H, respectively. Consequently, A_J_ (corresponding to the unsaturated protons (CH=CH) may be computed as a difference A_(I+J)_ − A_I_;
(iii) Since resonances A and B appear partially overlapped, they cannot be accurately integrated as separate signals; the integration was therefore performed according to the general rule (i.e., from baseline to baseline), leading to the integral of the envelope resonance (A+B).

The mean number of carbon atoms in the hydrocarbon chain (n_C_) and the average number of hydrogen atoms in the hydrocarbon chain (n_H_) can be computed as:

(3) $$n_{C}=M+2D+1$$

(4) $$n_{H}=2M+2D+3$$

leading to the mean formulae of the hydrocarbon chain (C_M+2D+1_H_2M+2D+3_) and of the triacylglycerol, i.e., C_6+3 (M+2D+1)_ H_5+3(2M+2D+3)_ O_6_.

As a consequence, the average molecular weight of TAGs becomes:

(5) $$M_{\mathrm{TAG}}=12\times \left[6+3\left(M+2D+1\right)\right]+1\times \left[5+3\left(2M+2D+3\right)\right]+16\times 6$$

The SV represents the amount of KOH (in mg) required for the saponification of 1 g of fat [15]. Therefore, SV can be computed as:

(6) $$\mathrm{SV}\;(\mathrm{mg}\;\mathrm{KOH}/g\;\mathrm{fat})=3\times \nu \times 56\times 10^{3}$$

where ν represents the number of TAG moles *per* gram of fat (ν = 1/M_TAG_), while (3 ⨯ ν) is the number of moles of ester groups *per* gram of oil.

An example of SV calculation from ^1^H-NMR data is shown in the Supplementary Materials (Table S2).

The SV values for the SO-TB and RO-TB series (both determined by the method based on the ^1^H-NMR data and determined experimentally by the conventional ISO 3657:2013 method taken as reference) are presented in Table 2.

As reflected in Table 2, the values obtained based on the ^1^H-NMR data were close to the values determined by the conventional method, which reflects the accuracy of the calculation algorithm.

**Figure 3:**
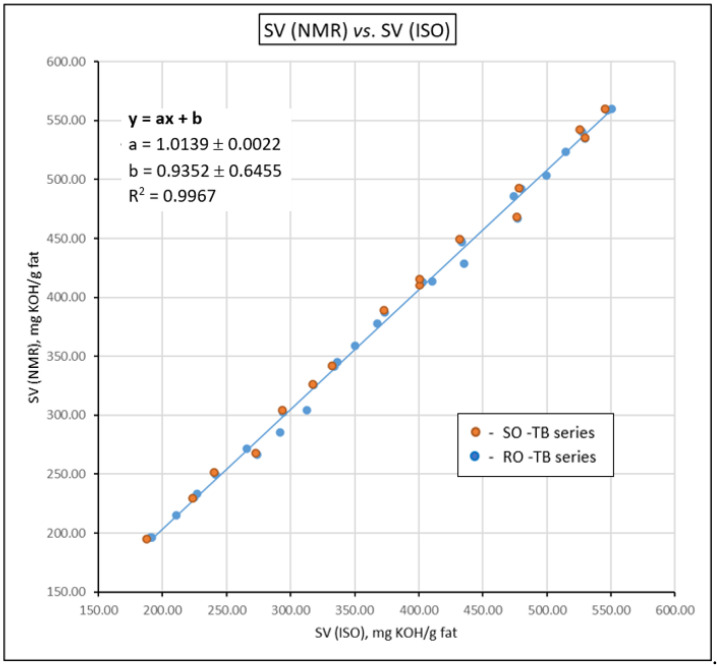
SV (NMR) plotted against the SV (ISO 3657:2013). Values for slope a and intercept b reported as the mean ± sd. The NMR experiments were performed in triplicate; ISO determinations were performed in duplicate.

The accuracy of the new method was assessed by calculating for each sample the SV (NMR) deviation from the SV (ISO), taken as a reference and expressed as percentages relative to the SV (ISO) (see details in Table S3). The mean percent deviation of SV (NMR) from SV (ISO) was found to be 2%, which stands for a robust NMR algorithm. The accuracy of the proposed method was also reflected by the SV (NMR) plotted against the SV (ISO) in Figure 3. The concordance between the values obtained by the NMR method and the titration values is reflected by values close to 1 for both the slope of the trendline (in the case of perfect concordance, tg α = 1, corresponding to an angle of 45°) and for the coefficient of correlation *R*^2^. As reflected from Figure 3, values close to 1 were obtained for the two parameters, indicating a good correlation between the two methods.

### 3.3. Determination of the SV for Edible Oils and Fats

Subsequently, the algorithm for determining the saponification value was applied to a series of commercial samples of vegetable oils and fats, butter, cheeses and spreadable fat mixtures (margarine type). The results are presented in Table 3.

As reflected from Table 3, there was agreement between the SVs calculated from the ^1^H-NMR data and the SVs determined through the wet (ISO 3657:2013) method. However, in the case of the oil and fat samples, the mean percent deviation of SV (NMR) from SV (ISO) was 3%, higher than in the case of the oil–TB series (2%), which may be due to the fact of their more complex composition compared to the binary mixtures.

Edible fats have variable SVs, depending on the species. As expected, vegetable oils, such as sunflower, soybean and rapeseed, had similar SVs, ranging from 194 to 196 mg KOH/g oil (as determined from the ^1^H-NMR data). These values are in agreement with the fatty acid composition consisting of C18 fatty acids (i.e., linoleic C18:2 and oleic C18:1 as the main constituents, various amounts of stearic C18:0 and linolenic acid C18:3 in small amounts) and modest amounts of C16:0 (palmitic) acid [21,22,23]. They are also in agreement with similar SVs reported in the literature [21]. On the other hand, lauric fats, such as coconut oil and palm fat, showed significantly higher SVs (mean values of 248.5 and 236.5 mg KOH/g oil, respectively) due to the fact of their specific fatty acid profiles rich in lauric (C12:0), myristic (C14:0) and myristoleic (C14:1) fatty acids. In the case of the coconut oil, its fatty acid profile is dominated by medium chain length fatty acids, with lauric acid ranging between 30 and 50% [24,25,26], while myristic was also reported in high levels (accounting for more than 20%) [24,25,26]. Palm fats are abundant in palmitic (C16:0) acid [25,27], with large amounts of lauric and myristic acids (especially palm kernel oil [3]). The high levels of C12 and C14 explain the marked increase in the SVs of coconut and palm fats compared to the rest of the vegetable oils.

In the case of dairy products (i.e., butter and cheese fats), the average saponification values were approximately 242 mg KOH/g fat in both cases. The SV results correlated with their particular fatty acid profile, containing mainly long chain (C14–C18) as well as important amounts of short (butyric, caproic) and medium (C8–C14) chain fatty acids [19,28]. It is worth mentioning that milk fats contain high amounts (up to 32.4% [29]) of palmitic acid (C16:0), whereas myristic (C14:0) and myristoleic (C14:1) acids occur in important amounts, accounting for more than 10–12% altogether [30,31]. Consequently—although belonging to the long chain fatty acids category—C14 fatty acids contributed to the global lowering of the average molecular weight of the triacylglycerols of milk fats compared to vegetable oils (mainly consisting of C16–C18 fatty acids). Altogether, the short and medium chain fatty acids, myristic and palmitic acid levels explain the high SV in the case of dairy products.

On the other hand, spreadable fat mixtures, the analyzed samples consisted of mixtures of butter with various amounts of vegetable fats. Given the variable composition of these samples (depending on the producers’ recipes), an average SV cannot be calculated. The spreadable fat mixtures have SV lower than those of butters and cheeses, due to the higher amounts of C16 and C18 fatty acids from the oils and fat ingredients of vegetal origin.

## 4. Perspectives

Milk fat is one of the most expensive ingredients in the food industry [19,32,33]; therefore, it may be subject to fraudulent practices such as its partial replacement with cheaper oils and fats. The addition of nondairy fats and oils to dairy fats will result in lower SVs. Of course, an altered butter or cheese fat composition would be difficult to detect through SVs if coconut oil (SV = ~249 mg KOH/g oil) combined with a common C16–C18 oil (such as sunflower, rapeseed or soybean oil, with SV = ~193 mg KOH/g oil) is used as an adulterant. On the other hand, except for the producing countries, coconut oil is an expensive commodity [34] in the rest of the regions (for example, in Europe), which makes it improbable as an adulterant. Consequently, SVs may be an indicator for dairy products adulteration with other fats and oils of nondairy origin. Therefore, further studies correlating the amount of vegetable fats added into dairy fats with the variation of the SV may lead to the rapid detection of adulterated dairy products.

## 5. Conclusions

All structural patterns of triacylglycerols were reflected as specific resonances in the ^1^H-NMR spectra of fats and oils. Chemometric equations leading to the mean molecular weight of triacylglycerol species may be derived from the integral values of the ^1^H-NMR signals, which may further be used to compute the number of moles of triacylglycerol species *per* gram of fat, which will further lead to the calculation of the SV, expressed as mg KOH/g of fat. Consequently, ^1^H-NMR spectroscopic data may be used to rapidly compute the saponification values of oils and fats based on the resonances associated with the fatty acyl chain lengths. The obtained ^1^H-NMR-based saponification values differed from the conventionally determined SVs by a mean percent deviation of 2.3%, which is sufficient to properly characterize various types of fats. Although the NMR method is more expensive than the official method, as was proven both by us and other groups, one can obtain more information (e.g., fatty acid composition and iodine number) in addition to the saponification value from the same NMR analysis in a very short time. Thus, for combined analyses both for advanced research and authentication purposes, SV by NMR is a valuable alternative.

## Figures and Tables

**Table 1:** Chemical shifts and peak assignment of ^1^H-NMR spectra of milk fats. Adapted with permission from Refs. [12,19]. Copyright 2004, *Eur. J. Lipid Sci. Technol*.; Copyright 2021, *J. Dairy Sci*.

Resonance *	δ (ppm)	Proton	Compound
**A**	0.85	-CH_2_-CH_2_-CH_2_-C*H*_3_	All acids except butyric acid and linolenic acid
**B**	0.96	-CH=CH-CH_2_-C*H*_3_	Linolenic acid
		-OOC-CH_2_-CH_2_-C*H*_3_	Butyric acid (**B’**)
**C**	1.24	-(C*H*_2_)_n_-	All fatty acids
**D**	1.64	-C*H*_2_-CH_2_-COO-	All fatty acids
**E**	2.02	-C*H*_2_-CH=CH-	All unsaturated fatty acids
**F**	2.26	-C*H*_2_-COO-	All fatty acids
**G**	2.76	-CH=CH-C*H*_2_-CH=CH-	n-6 (Linoleic) acid and n-3 (linolenic) acid
**H**	4.19	-C*H*_2_OCOR	H in the *sn-*1/3 position of the glycerol backbone
**I**	5.15	-C*H*OCOR	H in the *sn-*2 position of the glycerol backbone
**J**	5.29	-C*H*=C*H*-	All unsaturated fatty acids

* Letters from A–J correspond to specific resonances according to Figure 1.

**Table 2:** SVs determined from the ^1^H-NMR data and through the standard (i.e., ISO 3657:2013) method for the SO-TB and RO-TB series (95% confidence level).

SO-TB Series				RO-TB Series			
Sample	TB (%)	SV * (mg KOH/g Fat)		Sample	TB (%)	SV * (mg KOH/g Fat)	
		From ^1^H-NMR Data	According to ISO 3657:2013			From ^1^H-NMR Data	According to ISO 3657:2013
SO-TB-0	0	196 ± 2 ^aA^	190 ± 0 ^aB^	RO-TB-0	0	196 ± 4 ^aA^	192 ± 1 ^aA^
SO-TB-10	10	230 ± 4 ^bA^	225 ± 6 ^bA^	RO-TB-10	10	233 ± 3 ^bA^	227 ± 3 ^bA^
SO-TB-20	20	266 ± 2 ^cA^	274 ± 3 ^cA^	RO-TB-20	20	272 ± 2 ^pA^	266 ± 6 ^nA^
SO-TB-30	30	302 ± 2 ^dA^	294 ± 0 ^dB^	RO-TB-30	30	305 ± 4 ^dA^	312 ± 10 ^lA^
SO-TB-40	40	345 ± 3 ^eA^	336 ± 12 ^eA^	RO-TB-40	40	341 ± 2 ^eA^	334 ± 3 ^eA^
SO-TB-50	50	387 ± 2 ^fA^	374 ± 10 ^fA^	RO-TB-50	50	378 ± 2 ^qA^	367 ± 9 ^fA^
SO-TB-60	60	412 ± 1 ^gA^	403 ± 1 ^gB^	RO-TB-60	60	414 ± 3 ^gA^	411 ± 1 ^gA^
SO-TB-70	70	447 ± 1 ^hA^	434 ± 2 ^hB^	RO-TB-70	70	448 ± 1 ^hA^	433 ± 13 ^hA^
SO-TB-80	80	492 ± 2 ^iA^	480 ± 3 ^iB^	RO-TB-80	80	486 ± 3 ^rA^	474 ± 9 ^iA^
SO-TB-90	90	535 ± 3 ^jA^	530 ± 8 ^jB^	RO-TB-90	90	523 ± 2 ^sA^	515 ± 0 ^mB^
SO-TB-100	100	559 ± 2 ^kA^	547 ± 2 ^kB^	RO-TB-100	100	560 ± 3 ^kA^	551 ± 12 ^kA^
SO-TB-15	15	250 ± 3 ^lA^	241 ± 3 ^bA^	RO-TB-5	5	215 ± 2 ^tA^	211 ± 0 ^oA^
SO-TB-35	35	326 ± 3 ^mA^	318 ± 4 ^lA^	RO-TB-25	25	286 ± 2 ^uA^	292 ± 3 ^dA^
SO-TB-55	55	413 ± 1 ^gA^	403 ± 5 ^gA^	RO-TB-45	45	359 ± 3 ^vA^	350 ± 4 ^pA^
SO-TB-75	75	467 ± 3 ^nA^	477 ± 4 ^iA^	RO-TB-65	65	429 ± 2 ^wA^	435 ± 4 ^hA^
SO-TB-95	95	540 ± 2 ^oA^	527 ± 13 ^mA^	RO-TB-85	85	503 ± 3 ^xA^	499 ± 1 ^qA^

^a–x^ Means with different letters within a column are significantly different (*p* < 0.05). ^A, B^ Means with different letters within a row are significantly different (*p* < 0.05). * Determined in triplicate (NMR method) and in duplicate (ISO method); values are reported as the mean ± sd.

**Table 3:** SVs determined from ^1^H-NMR data and through the standard (ISO 3657:2013) method for a series of edible fats and oils (95% confidence level).

No.	Sample	SV * (mg KOH/g Fat)	
		From ^1^H-NMR Data	According to ISO 3657:2013
Sunflower oil			
1	Sunflower oil 1	194 ± 2 ^aA^	188 ± 2 ^aA^
2	Sunflower oil 2	195 ± 1 ^aA^	189 ± 2 ^aA^
3	Sunflower oil 3	194 ± 1 ^aA^	188 ± 3 ^aA^
4	Sunflower oil 4	196 ± 1 ^aA^	188 ± 3 ^aA^
5	Sunflower oil 5	195 ± 1 ^aA^	189 ± 2 ^aA^
Rapeseed oil			
6	Rapeseed oil 1	196 ± 1 ^aA^	188 ± 3 ^aB^
7	Rapeseed oil 2	196 ± 1 ^aA^	188 ± 2 ^aB^
8	Rapeseed oil 3	194 ± 1 ^aA^	188 ± 1 ^aB^
9	Rapeseed oil 4	195 ± 1 ^aA^	188 ± 2 ^aB^
Soybean oil			
10	Soybean oil 1	195 ± 2 ^aA^	189 ± 2 ^aB^
11	Soybean oil 2	193 ± 2 ^aA^	188 ± 2 ^aA^
12	Soybean oil 3	194 ± 1 ^aA^	187 ± 2 ^aB^
13	Soybean oil 4	195 ± 1 ^aA^	188 ± 2 ^aB^
14	Soybean oil 5	194 ± 1 ^aA^	188 ± 3 ^aA^
Coconut oil			
15	Coconut oil 1	249 ± 1 ^aA^	240 ± 3 ^aB^
16	Coconut oil 1	248 ± 1 ^aA^	239 ±1 ^aB^
Palm fat			
17	Palm fat 1	236 ± 1 ^aA^	230 ± 2 ^aA^
18	Palm fat 2	237 ± 1 ^aA^	230 ± 2 ^aB^
Butter			
19	Butter 1	242 ± 2 ^aA^	232 ± 1 ^aB^
20	Butter 2	245 ± 2 ^aA^	234 ± 1 ^aB^
21	Butter 3	245 ± 1 ^aA^	235 ± 1 ^aB^
22	Butter 4	239 ± 1 ^abA^	231 ± 2 ^aB^
23	Butter 5	241 ± 1 ^abA^	231 ± 1 ^aB^
Spreadable fat mixtures **			
24	Spreadable fat mixture 1	228 ± 1 ^aA^	217 ± 2 ^aB^
25	Spreadable fat mixture 2	206 ± 2 ^bA^	196 ± 1 ^bB^
26	Spreadable fat mixture 3	222 ± 2 ^cA^	217 ± 1 ^aA^
27	Spreadable fat mixture 4	224 ± 2a ^acA^	218 ± 1 ^aB^
Cheese			
28	Cheese 1	239 ± 2 ^aA^	231 ± 2 ^aB^
29	Cheese 2	242 ± 1 ^aA^	234 ± 1 ^aB^
30	Cheese 3	244 ± 2 ^baA^	237 ± 1 ^baB^
31	Cheese 4	238 ± 1 ^aA^	231 ± 2 ^aB^
32	Cheese 5	241 ± 2 ^aA^	233 ± 3 ^aA^
33	Cheese 6	241 ± 1 ^aA^	234 ± 1 ^aB^
34	Cheese 7	244 ± 2 ^bA^	237 ± 1 ^baB^
35	Cheese 8	244 ± 1 ^bA^	237 ± 2 ^baB^
36	Cheese 9	239 ± 1 ^aA^	233 ± 2 ^aB^

^a–c^ Means with different letters within a column are significantly different (*p* < 0.05). ^A, B^ Means with different letters within a row are significantly different (*p* < 0.05). * Determined in triplicate (NMR method) and in duplicate (ISO method), respectively; values reported as the mean ± sd. ** Variable composition (various amounts of butter and different vegetable oils).

## Data Availability

Data is contained within the article or supplementary material.

## Supplementary Materials

The following are available online at https://www.mdpi.com/article/10.3390/foods11101466/s1, Examples of the SV algorithm’s application—Table S1: Reference intervals for resonance integration; Table S2: 1H-RMN integral values for the UR-TB-20 sample; Table S3: Assessment of the accuracy of the NMR method; Table S4: Influence of various delays on the calculated SV.

## References

[1] Li Y., Watkins B.A., Wrolstad R.E. (2001). Unit D1.4: Oil Quality Indices. Current Protocols in Food Analytical Chemistry.

[2] Toscano G., Riva G., Foppa Pedretti E., Duca D. (2012). Vegetable oil and fat viscosity forecast models based on iodine number and saponification number. Biomass Bioenergy.

[3] Naksuk A., Sabatini D.A., Tongcumpou C. (2009). Microemulsion-based palm kernel oil extraction using mixed surfactant solutions. Ind. Crops Prod..

[4] Kilic B., Ozer C.O. (2019). Potential use of interesterified palm kernel oil to replace animal fat in frankfurters. Meat Sci..

[5] Marina A.M., Che Man Y.B., Nazimah S.A.H., Amin I. (2009). Chemical properties of virgin coconut oil. J. Am. Oil Chem. Soc..

[6] Sbihi H.M., Nehdi I.A., Tan C.P., Al-Resayes S.I. (2015). Characteristics and fatty acid composition of milk fat from Saudi Aradi goat. Grassas y Aceites.

[7] Salem E.R., Awad R.A., El Batawy O.I. (2019). Detection of Milk Fat Adulteration with Coconut Oil Depending on Some Physical and Chemical Properties. Int. J. Dairy Sci..

[8] Putri A.R., Rohman A., Setyaningsih W., Riyanto S. (2020). Determination of acid, peroxide, and saponification value in patin fish oil by FTIR spectroscopy combined with chemometrics. Food Res..

[9] Alexandri E., Ahmed R., Siddiqui H., Choudhary M.I., Tsiafoulis C.G., Gerothanassis I.P. (2017). High Resolution NMR Spectroscopy as a Structural and Analytical Tool for Unsaturated Lipids in Solution. Molecules.

[10] Yeung D.K.W., Lam S.L., Griffith J.F., Chan A.B.W., Chen Z., Tsang P.H., Leung P.C. (2008). Analysis of bone marrow fatty acid composition using high-resolution proton NMR spectroscopy. Chem. Phys. Lipids.

[11] Siudem P., Zielinska A., Paradowska K. (2022). Application of ^1^H NMR in the study of fatty acids composition of vegetable oils. J. Pharm. Biomed. Anal..

[12] Knothe G., Kenar J.A. (2004). Determination of the fatty acid profile by ^1^H-NMR Spectroscopy. Eur. J. Lipid Sci. Technol..

[13] Chira N.-A., Todasca M.-C., Nicolescu A., Rosu A., Nicolae M., Rosca S.-I. (2011). Evaluation of the computational methods for determining vegetable oils composition using ^1^H-NMR spectroscopy. Rev. Chim..

[14] Skiera C., Steliopoulos P., Kuballa T., Diehl B., Holzgrabe U. (2014). Determination of free fatty acids in pharmaceutical lipids by ^1^H NMR and comparison with the classical acid value. J. Pharm. Biomed..

[15] (2004). Cheese and Processed Cheese Products—Determination of Fat Content—Gravimetric Method (Reference method).

[16] Shahidi F., Whitaker J. (2001). Unit 1.1: Extraction and Measurement of Total Lipids. Current Protocols in Food Analytical Chemistry.

[17] (2013). Animal and Vegetable Fats and Oils—Determination of Saponification Value.

[18] Stavarache C., Nicolescu A., Duduianu C., Ailiesei G.L., Balan-Porcarasu M., Cristea M., Macsim A.-M., Popa O., Stavarache C., Hirtopeanu A. (2022). A real-life reproducibility assessment for NMR metabolomics. Diagnostics.

[19] Hanganu A., Chira N.-A. (2021). When detection of dairy food fraud fails: An alternative approach through proton nuclear magnetic resonance spectroscopy. J. Dairy Sci..

[20] Deleanu C., Enache C., Caproiu M.T., Cornilescu G., Hirtopeanu A. (1994). Esteri metilici ai acizilor grasi. Compusi etalon pentru atributia semnalelor in spectrele RMN de rezolutie inalta ale uleiurilor comestibile. Rev. Chim..

[21] Kuang G., Du Y., Lu S., Wang Z., Zhang Z., Fan X., Bilal M., Cui J., Jia S. (2022). Silica@lipase hybrid biocatalysts with superior activity by mimetic biomineralization in oil/water two-phase system for hydrolysis of soybean oil. LWT.

[22] Kampa J., Frazier R., Rodriguez-Garcia J. (2022). Physical and Chemical Characterisation of Conventional and Nano/Emulsions: Influence of Vegetable Oils from Different Origins. Foods.

[23] Wang J., Han Y., Wang X., Li Y., Wang S., Gan S., Dong G., Chen X., Wang S. (2022). Adulteration detection of Qinghai-Tibet Plateau flaxseed oil using HPLC-ELSD profiling of triacylglycerols and chemometrics. LWT.

[24] Figueiredo P.S., Martins T.N., Ravaglia L.M., Alcantara G.B., Guimarães R.d.C.A., Freitas K.d.C., Nunes Â.A., de Oliveira L.C.S., Cortês M.R., Michels F.S. (2022). Linseed, Baru, and Coconut Oils: NMR-Based Metabolomics, Leukocyte Infiltration Potential In Vivo, and Their Oil Characterization. Are There Still Controversies?. Nutrients.

[25] Dorni C., Sharma P., Saikia G., Longvah T. (2018). Fatty acid profile of edible oils and fats consumed in India. Food Chem..

[26] Kamath R., Basak S., Gokhale J. (2022). Recent trends in the development of healthy and functional cheese analogues-a review. LWT.

[27] Theam K.L., Islam A., Choo Y.M., Taufiq-Yap Y.H. (2015). Biodiesel from low cost palm stearin using metal doped methoxide solid catalyst. Ind. Crops Prod..

[28] Faccia M., Natrella G., Gambacorta G., Trani A. (2022). Cheese ripening in nonconventional conditions: A multiparameter study applied to Protected Geographical Indication Canestrato di Moliterno cheese. J. Dairy Sci..

[29] Salas-Valerio W.F., Aykas D.P., Hatta Sakoda B.A., Ludena-Urquizo F.E., Ball C., Plans M., Rodriguez-Saona L. (2022). In-field screening of trans-fat levels using mid- and near-infrared spectrometers for butters and margarines commercialized in the Peruvian market. LWT.

[30] Silva C.C.G., Silva S.P.M., Prates J.A.M., Bessa R.J.B., Rosa H.J.D., Rego O.A. (2019). Physicochemical traits and sensory quality of commercial butter produced in the Azores. Int. Dairy J..

[31] Wilms J.N., Hare K.S., Fischer-Tlustos A.J., Vahmani P., Dugan M.E.R., Leal L.N., Steele M.A. (2022). Fatty acid profile characterization in colostrum, transition milk, and mature milk of primi- and multiparous cows during the first week of lactation. J. Dairy Sci..

[32] Oduro A.F., Saalia F.K., Adjei M.Y.B. (2021). Sensory Acceptability and Proximate Composition of 3-Blend Plant-Based Dairy Alternatives. Foods.

[33] De Meneses R.B., Monteiro M.L.G., dos Santos F.F., da Rocha-Leão M.H.M., Conte-Junior C.A. (2021). Sensory Characteristics of Dairy By-Products as Potential Milk Replacers in Ice Cream. Sustainability.

[34] Amit, Jamwal R., Kumari S., Dhaulaniya A.S., Balan B., Kelly S., Cannavan A., Singh D.K. (2020). Utilizing ATR-FTIR spectroscopy combined with multivariate chemometric modelling for the swift detection of mustard oil adulteration in virgin coconut oil. Vib. Spectrosc..

