# Supplementary material for: Saponification Value of Fats and Oils as Determined from 1H-NMR Data: The Case of Dairy Fats
Source: Foods. 2022 May 18;11(10):1466. doi: 10.3390/foods11101466 (PMC9140812; doi:10.3390/foods11101466)
Supplement: Supplementary file 1 [file foods-11-01466-s001.zip › foods-1705664-supplementary.pdf]

Supplementary material

1. Reference intervals for resonance integration

Aiming to eliminate operator errors, fixed integration limits were used to obtain the integration values. In addition, the integrals of all F signals in the analyzed samples were calibrated to the value of 2,000, and the rest of the integrals were automatically calculated by the processing program (MestReNova, Mestrelab Research) accordingly. In Table S1, the intervals used to obtain the integrals are presented.

Table S1. Reference intervals for resonance integration

**Resonance***	**δ, ppm**
(A+B)	0.75 – 0.99
C	1.14 – 1.40
D	1.47 – 1.72
E	1.86 – 2.12
F	2.12 – 2.37
G	2.66 – 2.83
H	3.95 – 4.35
(I+J)	5.13 – 5.43

* Letters A-J according to the assignment of ^{1}H-NMR resonances presented in Table 1 and Figure 1.

2. Example of SV algorithm application

The calculation of the average molecular weight for the binary mixture RO-TB-20 (rapeseed oil - tributyrin 20%) is presented for example. The ^{1}H-NMR values of the integrals are presented in Table S2.

Table S2. ^{1}H-NMR integral values for RO-TB-20 sample

**^{1}H-NMR integral values***								
**Sample**	$A_{(A+B)}$	A_C	A_D	A_E	A_F	A_G	A_H	$A_{(I+J)}$
**RO-TB-20**	3.033	10.192	2.009	2.083	2	0.432	1.349	1.782

**RO-TB-20**	3.033	10.192	2.009	2.083	2	0.432	1.349	1.782

* Letters A-J according to the assignment of ^{1}H-NMR resonances presented in Table 1 and Figure 1.

$$M = \frac{3}{2} \cdot \frac{10.192 + 2.009 + 2.083 + 2 + 0.432}{3.033} = 8.267$$

$$D = \frac{3}{2} \cdot \frac{1.782 - \frac{1.349}{4}}{3.033} = 0.715$$

$n_C = 8.267 + 2 \cdot 0.715 + 1 = 10.696$ (mean number of C atoms in the alkyl chain)

$n_H = 2 \cdot 8.267 + 2 \cdot 0.715 + 3 = 20.963$ (mean number of H atoms in the alkyl chain)

The mean number of carbon atoms (n_C) and the average number of hydrogen atoms (n_H) in the triacylglycerol are computed as:

$$n_{C(TAG)} = 6 + 3 \cdot 10.969 = 38.09$$

$$n_{H(TAG)} = 5 + 3 \cdot 20.963 = 67.89$$

The mean formulae of the triacylglycerols will result as $C_{38.09}H_{67.89}O_6$, with the corresponding molecular weight:

$$M_{TG} = 12 \cdot 38.09 + 1 \cdot 67.89 + 16 \cdot 6 = 620.949$$

SV calculations:

$\nu = \frac{1}{M_{TAG}} = \frac{1}{620.949} = 1.61 \cdot 10^{-3}$ moles triacylglycerols/g fat

$\nu_{-CO-O-} = 3 \cdot \nu = 3 \cdot 1.61 \cdot 10^{-3} = 4.831 \cdot 10^{-3}$ moles ester groups/g fat

$SV = 3 \cdot \nu \cdot 56 \cdot 10^3 = 4.831 \cdot 10^{-3} \cdot 56 \cdot 10^3 =$ 271 mg KOH/g fat

3. Assessment of the method accuracy

The accuracy of the new method was assessed by calculating for each sample the SV (NMR) deviation from the SV (ISO), taken as a reference and expressed as percentages relative to

the SV (ISO) (see details in Table S2). The mean percent deviation of SV (NMR) from SV (ISO) was found 2.3%, which stands for a robust NMR algorithm.

Table S3. Assessment of the accuracy of the NMR method

Series	Sample	SV (NMR)	SV (ISO)	Deviation SV(NMR) from SV(ISO)	% Dev. against SV (ISO)	% Dev. as a module
				(5)=(3)-(4)	(6)=|(5)|	(7)=(6)×100/(4)
(1)	(2)	(3)	(4)	(5)	(6)	(7)
SO-TB series	SO-TB-0	196	190	6	6	3.2
	SO-TB-10	230	225	5	5	2.2
	SO-TB-20	266	274	-8	8	2.9
	SO-TB-30	302	294	8	8	2.7
	SO-TB-40	345	336	9	9	2.7
	SO-TB-50	387	374	13	13	3.5
	SO-TB-60	412	403	9	9	2.2
	SO-TB-70	447	434	13	13	3.0
	SO-TB-80	492	480	12	12	2.5
	SO-TB-90	535	530	5	5	0.9
	SO-TB-100	559	547	12	12	2.2
	SO-TB-15	250	241	9	9	3.7
	SO-TB-35	326	318	8	8	2.5
	SO-TB-55	413	403	10	10	2.5
	SO-TB-75	467	477	-10	10	2.1
	SO-TB-95	540	527	13	13	2.5
RO-TB series	RO-TB-0	196	192	4	4	2.1
	RO-TB-10	233	227	6	6	2.6
	RO-TB-20	272	266	6	6	2.3
	RO-TB-30	305	312	-7	7	2.2
	RO-TB-40	341	334	7	7	2.1
	RO-TB-50	378	367	11	11	3.0
	RO-TB-60	414	411	3	3	0.7
	RO-TB-70	448	433	15	15	3.5
	RO-TB-80	486	474	12	12	2.5
	RO-TB-90	523	515	8	8	1.6
	RO-TB-100	560	551	9	9	1.6
	RO-TB-5	215	211	4	4	1.9
	RO-TB-25	286	292	-6	6	2.1
	RO-TB-45	359	350	9	9	2.6
	RO-TB-65	429	435	-6	6	1.4
	RO-TB-85	503	499	4	4	0.8

**Average**	**2.3**

4. Influence of various delays on the calculated SV

The influence of various delays on the calculated SV has been checked on a rapeseed oil sample. The corresponding integral values and the calculated SV are presented in Table S4.

Table S4. Influence of various delays on the calculated SV*

**D1 [sec]**	**Int. A+B**	**Int. C**	**Int. D**	**Int. E**	**Int. F**	**Int. G**	**Int. H**	**Int. I+J**	**SV**	**SV (formatted)**
**0.5**	100.000	576.407	64.286	115.278	62.965	22.717	41.797	89.619	199.79	**200**
**1**	100.000	575.227	64.077	115.015	62.824	22.674	41.619	89.555	200.07	**200**
**2**	100.000	572.998	63.741	114.543	62.517	22.558	41.416	89.313	200.65	**201**
**3**	100.000	575.057	64.226	115.098	62.854	22.725	41.693	89.722	200.01	**200**
**4**	100.000	572.986	63.871	114.640	62.553	22.603	41.513	89.440	200.57	**201**
**5**	100.000	573.870	64.041	114.849	62.702	22.667	41.595	89.616	200.31	**200**
**6**	100.000	573.073	63.909	114.687	62.583	22.626	41.526	89.515	200.52	**201**
**7**	100.000	572.161	63.739	114.481	62.448	22.574	41.453	89.377	200.78	**201**
**8**	100.000	572.289	63.771	114.505	62.459	22.578	41.462	89.406	200.74	**201**
**9**	100.000	572.123	63.753	114.474	62.450	22.571	41.458	89.386	200.78	**201**
**10**	100.000	572.732	63.863	114.622	62.533	22.609	41.501	89.486	200.61	**201**
**11**	100.000	571.866	63.719	114.401	62.403	22.547	41.422	89.355	200.85	**201**
**12**	100.000	572.864	63.912	114.656	62.567	22.623	41.518	89.522	200.56	**201**
**13**	100.000	572.814	63.882	114.643	62.562	22.617	41.512	89.510	200.58	**201**
**14**	100.000	571.757	63.698	114.397	62.389	22.554	41.424	89.343	200.88	**201**
**15**	100.000	572.801	63.870	114.650	62.562	22.626	41.515	89.513	200.58	**201**
**16**	100.000	572.147	63.787	114.491	62.468	22.583	41.458	89.413	200.76	**201**
**18**	100.000	571.397	63.639	114.295	62.340	22.519	41.386	89.282	200.98	**201**
**20**	100.000	571.898	63.733	114.444	62.428	22.563	41.440	89.371	200.83	**201**
**24**	100.000	573.327	63.994	114.758	62.655	22.650	41.554	89.610	200.42	**200**
**60**	100.000	572.701	63.857	114.590	62.545	22.598	41.496	89.502	200.61	**201**
**120**	100.000	572.135	63.753	114.461	62.445	22.564	41.459	89.407	200.78	**201**

* Letters A-J according to the assignment of ^{1}H-NMR resonances presented in Table 1 and Figure 1.
